# Bowel obstruction caused by an internal hernia that developed after laparoscopic subtotal colectomy: a case report

**DOI:** 10.1186/1752-1947-8-470

**Published:** 2014-12-29

**Authors:** Takefumi Yoshida, Tetsushi Kinugasa, Yousuke Oka, Tomoaki Mizobe, Hiroto Ishikawa, Naoki Mori, Taro Isobe, Eri Katayama, Yoshito Akagi

**Affiliations:** Department of Surgery, Kurume University School of Medicine, 67 Asahi-machi, Kurume-shi, Fukuoka-ken, 810-0023 Japan

**Keywords:** Laparoscopic surgery, Subtotal colectomy, Bowel strangulation, Internal hernia, Mesenteric closure

## Abstract

**Introduction:**

Laparoscopic surgery is a minimally invasive approach with good treatment outcomes and is currently the standard surgery for colorectal cancer in Japan. Mesenteric closure is considered unnecessary in laparoscopic colorectal surgery because it can damage the bowel and blood vessels. However, an internal hernia may develop if the mesentery is not repaired.

**Case presentation:**

We report a case of internal hernia in a 61-year-old male of Japanese ethnicity. The patient had advanced sigmoid colon cancer, early-stage transverse colon cancer, and multiple adenomatous polyposis, and underwent laparoscopically-assisted subtotal colectomy. Bowel obstruction developed six days postoperatively and did not improve with conservative treatment. Abdominal computed tomography detected an internal hernia, prompting emergency surgery in which the ileum protruding into the mesenteric defect and an anastomotic stricture were detected. Reanastomosis, mesentery closure, and ileostomy were performed after hernia repair.

**Conclusion:**

In this case, open surgery was necessary due to bowel obstruction after laparoscopic colectomy. This outcome indicated that mesenteric closure should have been performed. Thus, the benefits of mesenteric closure require assessment in future cases.

## Introduction

Laparoscopic surgery for colon cancer yields treatment outcomes equivalent to those in open surgery, and thus this approach has been rapidly adopted [1]. Laparoscopic surgery also benefits patients because it leaves a smaller scar than open surgery and is minimally invasive [2]. However, some negative effects can arise as a result of conditions unique to laparoscopic surgery, including rising intraperitoneal pressure due to pneumoperitoneum and steep head-up or head-down positions, and there have been reports of development of internal hernias if the mesentery is not repaired [3–8]. The mesenteric defect is typically closed after open surgery, but this is generally not considered to be necessary after laparoscopic surgery [6]. Here, we report a case of bowel obstruction caused by an internal hernia that developed after laparoscopic subtotal colectomy without mesenteric repair.

## Case presentation

**Figure 1:**
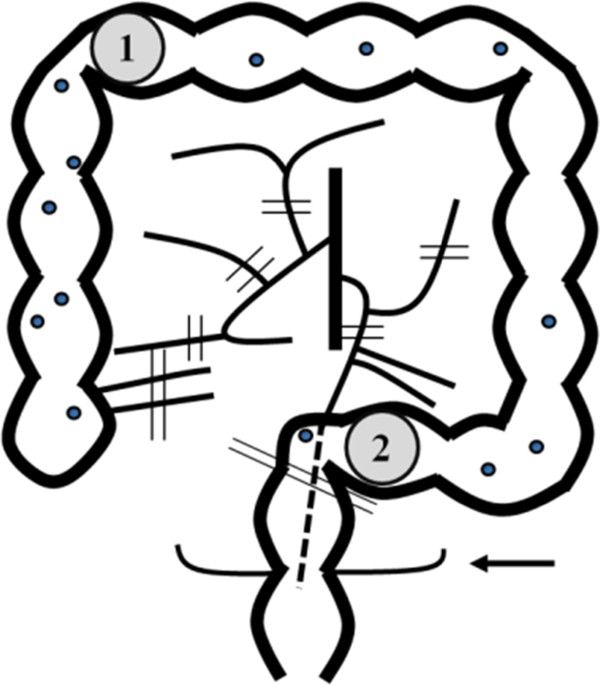
**Schema showing locations of cancers and adenomas, and blood vessels and bowel resection sites.** ① ②: carcinomas; small circles: polyps; parallel lines: resection lines; arrow: peritoneal reflection.

**Figure 2:**
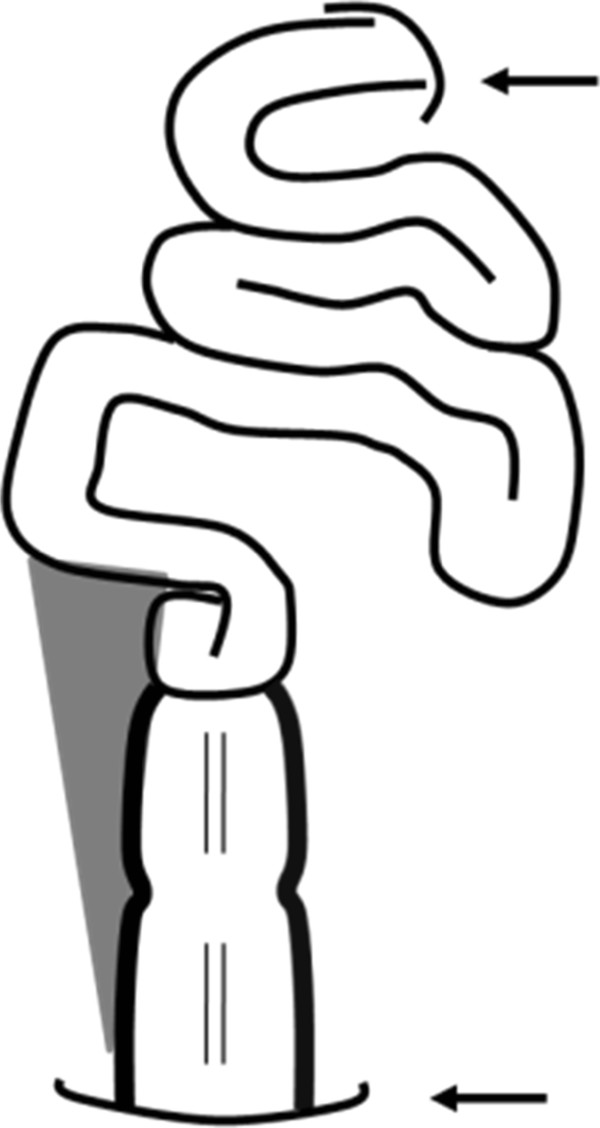
**Schema after side-to-end coloanal anastomosis.** Upper arrow: Treitz ligament; lower arrow: peritoneal reflection; gray zone indicates unrepaired space.

The patient was a 61-year-old male of Japanese ethnicity. He was 167cm tall, weighed 42kg, and had a body mass index (BMI) of 15kg/m^2^. He was admitted to our hospital to undergo surgery for multiple colorectal tumors. Colonoscopy revealed sigmoid colon cancer, an early cancer in the transverse colon, and several adenomas across the length of the entire colon (Figure 1). Laparoscopic subtotal colectomy was performed under general and epidural anesthesia, after informed consent was obtained from the patient. The inferior mesenteric artery and vein were resected distal the left colic artery branch. The right and left side of the colon and each mesentery and the hepatic and splenic flexure were mobilized after the area between the transverse colon and the greater omentum was resected. During detachment of the splenic flexure, all layers of the transverse colon were damaged and the intestinal contents flowed out. The ileocolic artery and vein, and the right colic and middle colic vessels were resected at each trunk. The rectum was detached up to the peritoneal reflection. Ten centimeters of the colon to the anal side of the sigmoid colon cancer was transected using a linear stapler (Echelon Flex™ Powered Endopath® Stapler 60; Ethicon, USA). After the colon was pulled out from the umbilical region and the ileum end was transected, the colon was excised. Side-to-end ileorectal anastomosis was performed using a circular stapler (Proximate* ILS CDH 29; Ethicon, USA) (Figure 2). Penrose drains were inserted below the left crus of the diaphragm and at the posterior surface of the anastomosis after the peritoneal cavity was washed with 3,000 cc of warm physiological saline. The small bowel was returned to its original position, antiadhesive material was placed inside the cavity, and the abdomen was closed. The duration of surgery was 8 h and 22 min, with 200 cc of blood loss.

**Figure 3:**
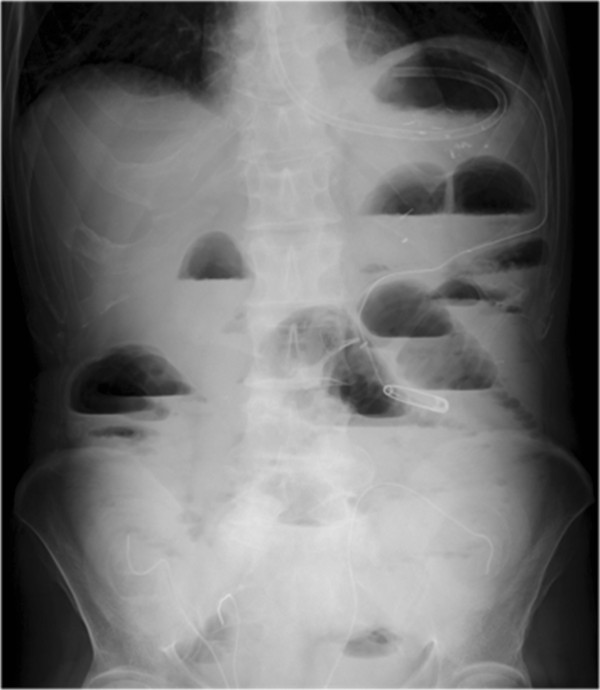
**Plain standing abdominal X-ray.** Niveau formation in the small intestine.

The final diagnosis was T2N0M0 stage I moderately differentiated adenocarcinoma for the sigmoid colon cancer, stage 0 (TisN0M0) well differentiated adenocarcinoma for the transverse colon cancer, and tubular adenomas (10 low grade adenomas, 3 high grade adenomas) for other polyps. Bowel obstruction developed on the sixth day after surgery (Figure 3) and did not improve, even after an ileus tube was inserted and conservative treatment was performed. He had fever and abdominal distension. Blood tests (Table 1) revealed an elevated inflammatory response, hypoalbuminemia, impaired liver function, and coagulation system abnormalities.

**Figure 4:**
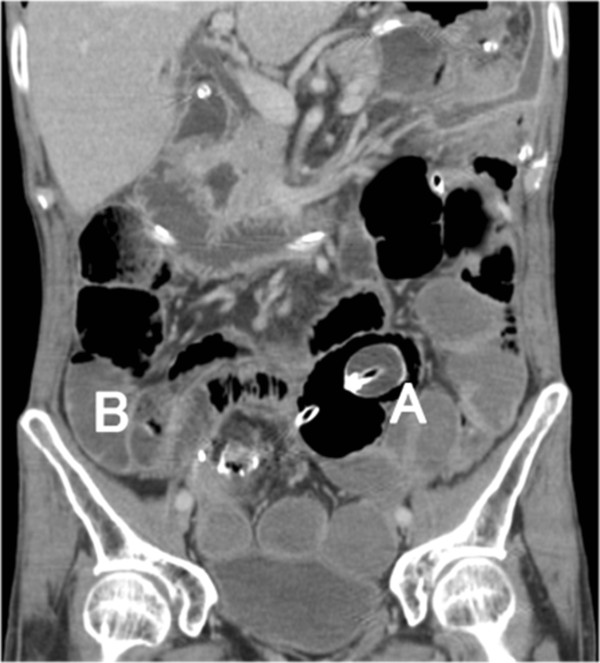
**Abdominal contrast computed tomography.** The tip of the ileus tube stops at the origin of the obstruction. The site of the obstruction appears to be the hernial orifice and internal herniation of the small bowel is apparent past that point. **A**: Ileus tube balloon, **B**: Internal herniation of the small bowel.

**Figure 5:**
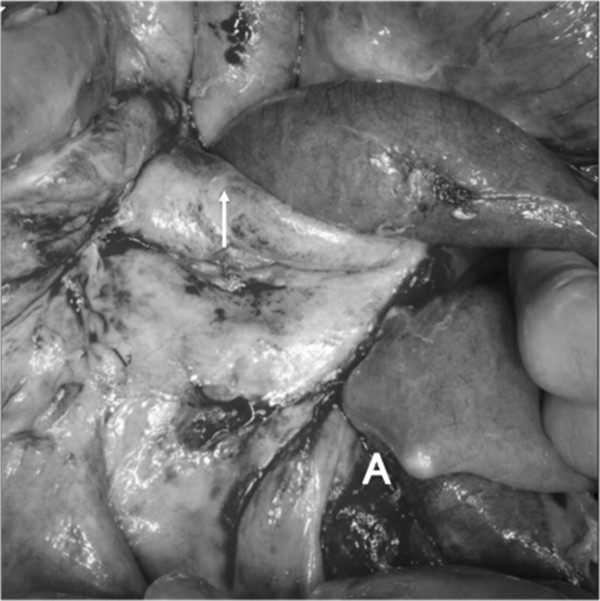
**The small bowel protruded into the opening of the hernia (** ***arrow*** **), which was formed by the mesenteric defect.** **A**: Tip of the ileus tube. The top of the image is the cranial side.

**Figure 6:**
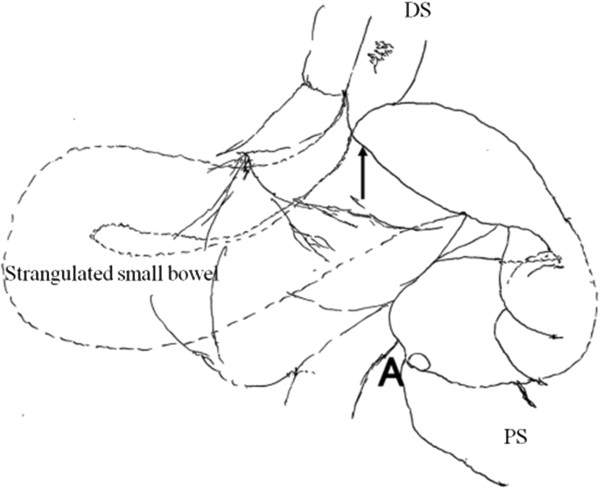
**Schema of Figure** 5 **.** Dotted line shows the protruding section of the small bowel. Arrow: hernial orifice; **A**: ileus tube; DS: distal side; PS: proximal side.

Emergency surgery was performed because bowel obstruction caused by internal hernia was suspected based on abdominal computed tomography (CT) findings (Figure 4). The upper part of the small bowel was dilated because the ileum had protruded into the mesenteric defect, leading to an internal hernia. The tip of the ileus tube had stopped at the oral side of the incarcerated herniated intestine (Figures 5 and 6). The area around the anastomosis had hardened and was determined to be an anastomotic stricture. The incarcerated herniated bowel was 30cm long, whereas the ileum to the oral side of the ileorectal anastomosis to the anal side was 40cm long. Re-anastomosis was performed after hernia repair because the situation was treated as a stenosis, even though circulation to the bowel of the ileorectal anastomosis was not interrupted. The mesenteric defect was closed using 3–0 Vicryl, and a Penrose drain was inserted into the posterior of the anastomosis. A loop ileostomy was them constructed. The patient began eating on the third day after emergency surgery and recovered with no major problems. He was discharged 28 days after surgery.

**Table 1:** **Blood test findings before emergency surgery at 6 days after subtotal colectomy**

Item	Value
**White blood cells WBC (/μL)**	**13800**
Red blood cells RBC (10^4^/μL)	333
Hemoglobin (Hb) (g/dL)	11.4
Hematocrit (Ht) (%)	31.4
Platelets (Plt) (10^4^/μL)	58.2
Albumin (Alb**)** (g/dL)	3.03
Total bilirubin (T.Bil**)** (mg/dL)	0.44
**Aspartate aminotransferase (AST) (U/L)**	**141**
**Alanine aminotransferase (ALT) (U/L)**	**140**
Lactic acid dehydrogenase (LD**)** (U/L)	215
Alkaline phosphatase (ALP**)** (U/L)	386
γ-Glutamyltranspeptidase (γ-GTP**)** (U/L)	81
Blood urea nitrogen (BUN**)** (mg/dL)	10
Creatinine (Cre**)** (mg/dL)	0.45
**Sodium (Na) (mmol/L)**	**126**
**Chloride (Cl) (mmol/L)**	**87**
Potassium (K**)** (mmol/L)	5.2
Amylase (Amy**)** (U/L)	79
**C-reactive protein test (CRP) (mg/dL)**	**8.26**
**Prothrombin time (PT) (%)**	**71**
**Activated partial thromboplastin time (APTT) (sec)**	**42.8**
**Fibrin and fibrinogen degradation product (FDP) (μg/mL)**	**21.2**

Data shown in bold are elevated from normal levels.

## Discussion

In recent years, there has been an increase in the number of laparoscopic surgeries for colorectal cancer in Japan, as shown by the results of a survey conducted by the Japan Society for Endoscopic Surgery [9]. Laparoscopic surgery has been proven to be comparable with open surgery and is becoming the standard method for colorectal cancer surgery [1, 2]. Aggregate data in Japan indicates that 82,291 patients with benign or malignant conditions of the small or large bowel underwent laparoscopic surgery from 2008 to 2011, of which 4,912 (6%) experienced a procedural accident, including 1,172 cases (1%) of bowel obstruction [9]. Bowel obstruction caused by an internal hernia after laparoscopic surgery has been reported in Roux-en-Y reconstruction [10–12], Nissen fundoplication [13], and various types of laparoscopic urogenital surgeries [14].

There are also several reports of internal hernia after laparoscopic colorectal cancer surgery [3–8]. Among these, Cabot *et al.* [6] observed 4 cases of internal hernia in 530 laparoscopic right colon surgeries (0.8%) and Trabaldo *et al.* [7] observed 5 cases in 436 laparoscopic left colon surgeries (1.14%). Only 2 deaths were reported in these studies [6, 7], but the individual risk is uncertain. A narrow defect hole (2–5cm) caused by incomplete closure may increase the risk of a symptomatic internal hernia [8]. Internal hernia in our patient might have developed due to the long duration of surgery; or may have been caused by protrusion of the bowel into the mesenteric defect due to the higher intraperitoneal pressure during postoperative bowel paresis or intraperitoneal infection. Given the low incidence of internal hernias, mesenteric closure may not be necessary for every patient. However, closure should be considered in bowel damage during surgery if postoperative bowel paresis is likely or if the patient has a thin physique, as for our patient. Masubuchi *et al.* [3] proposed that the mesenteric defect should be closed in thin patients because they are at risk for developing an internal hernia.

The current case shows that both frontal and transverse CT images are useful for diagnosis of internal hernias. Trabaldo *et al.* [7] also used CT scans for diagnosis. In our case, bowel obstruction arose from an internal hernia caused by the absence of repair of the mesentery after laparoscopic subtotal colectomy. Diagnosis with CT permitted subsequent performance of emergency surgery.

## Conclusion

We have reported a case of internal hernia that developed after laparoscopic surgery for colon cancer. This case suggests that the benefits of mesenteric closure after laparoscopic colorectal surgery should be examined further in patients with risk factors for development of internal hernia. In such patients, postoperative abdominal computed tomography may be useful for detection of a potential hernia.

## Consent

Written informed consent was obtained from the patient for publication of this case report and accompanying images. A copy of the written consent is available for review by the Editor-in-Chief of this journal.

## Abbreviations

BMI: Body mass index
CT: Computed tomography.
